# Graphene Oxide/Cholesterol-Substituted Zinc Phthalocyanine Composites with Enhanced Photodynamic Therapy Properties

**DOI:** 10.3390/ma16227060

**Published:** 2023-11-07

**Authors:** Fuat Erden

**Affiliations:** Department of Aeronautical Engineering, Sivas University of Science and Technology, 58000 Sivas, Türkiye; fuaterden@sivas.edu.tr

**Keywords:** photodynamic therapy, phthalocyanine, graphene oxide, cholesterol, singlet oxygen

## Abstract

In the present work, cholesterol (Chol)-substituted zinc phthalocyanine (Chol-ZnPc) and its composite with graphene oxide (GO) were prepared for photodynamic therapy (PDT) applications. Briefly, Chol-substituted phthalonitrile (Chol-phthalonitrile) was synthesized first through the substitution of Chol to the phthalonitrile group over the oxygen bridge. Then, Chol-ZnPc was synthesized by a tetramerization reaction of Chol-phthalonitrile with ZnCl_2_ in a basic medium. Following this, GO was introduced to Chol-ZnPc, and the successful preparation of the samples was verified through FT-IR, UV–Vis, ^1^H-NMR, MALDI-TOF MS, SEM, and elemental analysis. Regarding PDT properties, we report that Chol-ZnPc exhibited a singlet oxygen quantum yield ($\Phi_{∆}$) of 0.54, which is slightly lower than unsubstituted ZnPc. Upon introduction of GO, the GO/Chol-ZnPc composite exhibited a higher $\Phi_{∆}$, about 0.78, than that of unsubstituted ZnPc. Moreover, this enhancement was realized with a simultaneous improvement in fluorescence quantum yield ($\Phi_{F}$) to 0.36. In addition, DPPH results suggest low antioxidant activity in the composite despite the presence of GO. Overall, GO/Chol-ZnPc might provide combined benefits for PDT, particularly in terms of image guidance and singlet oxygen generation.

## 1. Introduction

Photodynamic therapy (PDT) is a noninvasive cancer treatment method with fewer side effects and many more advantages than chemotherapy, radiotherapy, and surgery. In PDT, a light source, oxygen in the human tissue, and a photosensitizer are used in conjunction to destroy cancerous cells [1,2,3]. Once a photosensitizer is activated with a red light, it transfers energy to the dissolved oxygen in the vicinity of the cancer cell to produce reactive oxygen species (ROS) [2,3]. Singlet oxygen is one of the most well-known molecular oxygen derivatives among ROS with high toxicity that can directly kill cancer cells [3,4]. This means that an ideal photosensitizer material should exhibit a high singlet oxygen quantum yield in addition to a high absorption peak in the wavelengths corresponding to the therapeutic window. In this sense, phthalocyanine (Pc) and its derivatives are regarded as second-generation photosensitizer materials, since they not only meet the above requirements, but also could reduce skin phototoxicity as compared to photofrin-based first-generation photosensitizers [1,5,6]. Particularly, zinc Pc (ZnPc) and its derivatives are receiving considerable interest for PDT applications thanks to their relatively high efficiencies to yield singlet oxygen, low toxicity, easy synthesis, ease in modifying their structure, and high stability [7,8,9,10,11,12,13]. However, their main drawback is their poor solubility in aqueous solutions as a result of their extended flat hydrophobic aromatic surface, which makes it difficult to transport them to the target cell in the human body [14,15].

One way to resolve this involves the encapsulation of Pcs in liposome-based nanoparticles for their targeted delivery [16,17,18,19,20,21,22]. In that case, liposomes release Pcs during lipid exchange with lipoproteins, and then Pcs are bound to lipoproteins, which carry cholesterol (Chol) through the bloodstream [23,24,25]. The binding of Pcs to lipoproteins, particularly to Chol-rich, low-density lipoproteins (LDL), results in the selective targeting of cancer cells [23,24,25,26,27,28,29]. As is known, cancer is the uncontrollable and abnormal growth of cells. Thus, cancer cells need excessive Chol levels for proliferation; hence, they express higher LDL receptors than noncancer cells [23,24,25,26,27]. This, in turn, results in the systematic and selective accumulation of Pcs in tumor cells through receptor-mediated endocytosis [29,30]. In this sense, previous reports indicate that the encapsulation of ZnPcs in liposomes in the presence of cholesterol (Chol) significantly improves their photodynamic activity [19,20,21,22]. Chol is an organic molecule, and its influence on cancer has been studied since it was first reported in the early 1900s [31,32,33]. It is known that the restriction of Chol could prevent the growth of cancer cells, and cancer cells could attract Chol through LDL receptors, as mentioned previously [34,35,36].

Apart from this, it is known that the composites of Pc with low-dimensional carbon materials could enhance the charge transfer properties through π–π stacking. Particularly, graphene oxide (GO) is receiving considerable interest as a constituent material in such composite structures due to its oxygen-containing functional groups and high photothermal conversion efficiency [37,38,39,40,41]. In this context, various GO/Pc composites have been investigated for PDT, combined photothermal/PDT, combined sonodynamic/PDT, combined drug delivery/PDT, and image-guided photothermal/PDT applications, with promising results [41,42,43,44,45,46,47,48]. For instance, Rocha et al. reported that GO/ZnPc and GO/ZnPc(NO_2_)_4_ exhibited singlet oxygen quantum yields of about 0.71 and 0.32 in dimethylformamide (DMF), respectively, of which both values were higher than that of the singlet oxygen quantum yields of ZnPc and ZnPc(NO_2_)_4_ in DMF, which were only about 0.56 and 0.25, respectively [42]. Further, it was reported that a similar situation was persistent in acetonitrile (MeCN), since GO/ZnPc and GO/ZnPc(NO_2_)_4_ exhibited singlet oxygen quantum yields of about 0.71 and 0.29 in MeCN, while ZnPc and ZnPc(NO_2_)_4_ displayed much lower performances with singlet oxygen quantum yields of 0.50 and 0.27, respectively [42].

Based on the above considerations, we propose that Chol-substituted ZnPc and its composite with GO might provide interesting properties for PDT applications. Accordingly, the present work involves the preparation of Chol-ZnPc through a tetramerization reaction of Chol-substituted phthalonitrile (Chol-phthalonitrile) with zinc chloride (ZnCl_2_). Then, GO/Chol-ZnPc composites were prepared with the self-assembly method through π–π interactions. The samples were characterized using FT-IR, UV–Vis, ^1^H-NMR, MALDI-TOF MS, SEM, and elemental analysis, and both fluorescence quantum yield (Φ*_F_*) and singlet oxygen quantum yield (Φ_∆_) of Chol-ZnPc and GO/Chol-ZnPc were studied. Also, the antioxidant potency of the GO/Chol-ZnPc was determined considering the presence of GO in the composite, and the PDT potentials of the as-prepared materials were discussed. 

## 2. Materials and Methods

### 2.1. Materials

Chol (≥99%), 4-nitrophthalonitrile (99%), ZnCl_2_ (≥98%), potassium carbonate (≥98%), dimethyl sulfoxide (DMSO) (≥99.5%), chloroform (anhydrous, ≥99%), hexane (anhydrous, 95%), 1-pentanol (≥99%), 1,8-diazabicyclo[5.4.0]undec-7-ene (DBU) (≥99.0%), and methanol (anhydrous, 99.8%) were purchased from Sigma Aldrich (Saint Louis, MO, USA) and used as received. GO was obtained from Nanografi Co., Ltd. (Ankara, Turkey), and used as received. Deionized (DI) water was used throughout the experiments.

### 2.2. Synthesis of Chol-ZnPc

**Figure 1:**
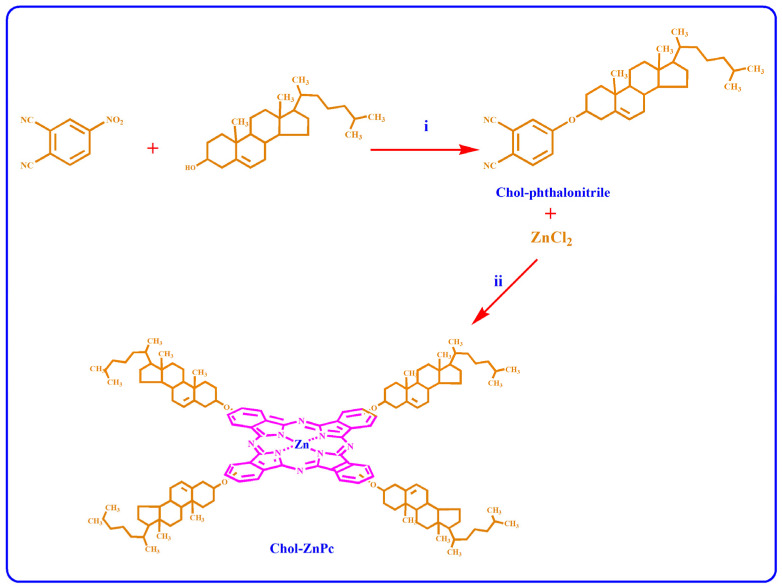
Synthesis of Chol-substituted phthalonitrile (i) and Chol-ZnPc (ii).

First, we synthesized Chol-phthalonitrile, which was later used in the synthesis of Chol-ZnPc. For this, 1 g of 4-nitrophthalonitrile was mixed with 2.46 g of Chol in 25 mL DMSO. Then, 2.4 g of potassium carbonate was gradually introduced to the solution at room temperature over a 2 h period. The mixture was stirred at room temperature for 5 days. Following this, the solvent was removed in an evaporator, and the residue was dis-solved in chloroform and filtered. The reaction products were purified by column chromatography on silica gel using a mixture of chloroform and hexane (6:1). The yield for the synthesis of Chol-phthalonitrile was calculated as 26%, and the melting point of Chol-phthalonitrile was measured as 186 °C. For the synthesis of Chol-ZnPc, 100 mg of light yellow powder of as-synthesized Chol-phthalonitrile was mixed with 6.8 mg of ZnCl_2_ in 1 mL 1-pentanol in the presence of DBU. Then, the reaction was performed at 185 °C for 22 h. After cooling, the mixture was precipitated with methanol, filtered, and dried in a vacuum. The obtained dark green powders were then dissolved in chloroform and filtered. The residue was further purified by column chromatography over a silica gel eluted with a 9:2 mixture of chloroform and methane. The yield for the synthesis of Chol-ZnPc was calculated as 15%, and the melting point of Chol-ZnPc was found to be higher than 300 °C. The reactions are schematized in Figure 1.

Chol-phthalonitrile: Yield 770 mg (26%). Mp: 186 °C. ^1^H-NMR (400 MHz, CDCl_3_, 25 °C): δ = 8.70 (2H, Ar-H); 8.1 (1H, Ar-H); 5.3 (1H, Chol-H); 3.4 (1H, Chol-H); 2.2 (2H, Chol-H); 1.0 (3H, Chol-CH3); 0.9 (6H, Chol-CH3); 0.8 (3H, Chol-CH3); 0.7 (3H, Chol-CH3). FT-IR (KBr pellet) υ (cm^−1^) 3086; 3012; 2239; 1468; 731. Anal. Calc. for C_35_H_48_N_2_O: C, 81.98; H, 9.44; N, 5.46%, found: C, 81.05; H, 8.89; N, 5.70%.

Chol-ZnPc: Yield 41.0 mg (15%). Mp: >300 °C. ^1^H-NMR (400 MHz, CDCl_3_, 25 °C): δ = 8.1–7.1 (12H, Ar-H); 5.5 (1H, Chol-H); 3.6 (1H, Chol-H); 2.5–0.5 (43H, Chol-H). UV–Vis (DMSO) λ_max_/nm 724. 703, 639, 368. FT-IR (KBr pellet) υ (cm^−1^) 3056; 2926; 2860; 1645; 1608; 1522; 1491; 1444; 1398; 1315; 1261; 1144; 1101; 930; 768; 700. Anal. Calc. for C_140_H_120_N_8_O_4_Zn: C, 82.27; H, 5.92; N, 5.48%, found: C, 80.12; H, 5.27; N, 5.52%. MALDI-TOF MS *m*/*z*: 2045 [M+H]^+^.

### 2.3. Preparation of GO/Chol-ZnPc Composites

**Figure 2:**
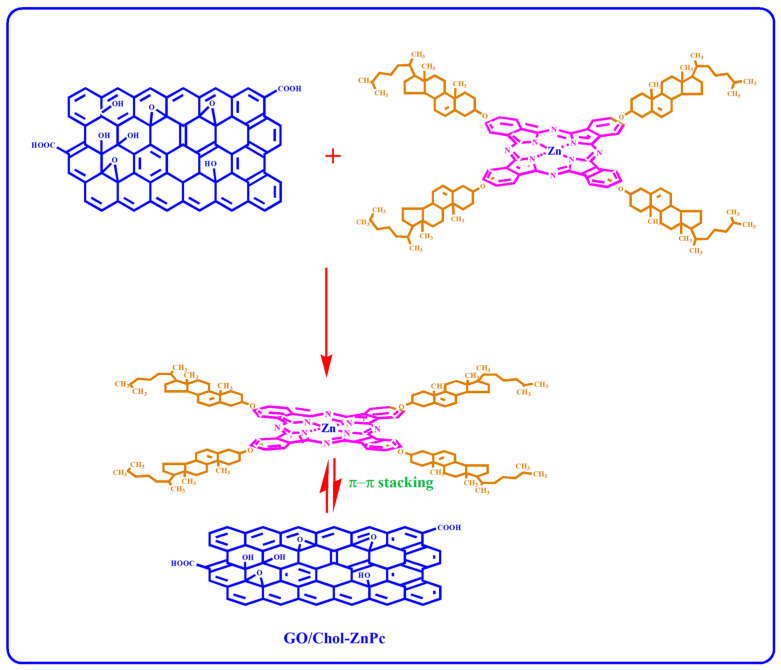
Preparation of GO/Chol-ZnPc composites.

GO (2 µg/mL) was dispersed in DMSO at room temperature by using a Bandelin HD 2070 ultrasonic homogenizer (Berlin, Germany). At the same time, Chol-ZnPc was also prepared in DMSO (5 µg/mL), and then introduced to the GO-DMSO mixture. The resultant solution was further sonicated for 25 min at room temperature. Then, the GO/Chol-ZnPc composites were filtered, washed with DI water, and then dried in vacuum oven until a constant weight was reached. The weight percentage of GO (based on the feed composition) in the composite was ~29%. The process is schematized in Figure 2.

### 2.4. Characterization

Absorption and fluorescence spectra of the samples were measured on a Shimadzu UV-1800 spectrophotometer (Kyoto, Japan) and Agilent G9800A fluorescence spectrophotometer (Santa Clara, CA, USA) in DMSO, respectively. As is known, concentration has an effect on such measurements [49,50], and it was kept at 5 μg/mL in the present work. Fourier-transform infrared (FT-IR) spectra were recorded using a Bruker Tensor II FT-IR spectrophotometer (Ettlingen, Germany). Elemental analysis was performed using an LECO elemental analyzer (Saint Joseph, MI, USA). The morphologies of the as-prepared samples were examined on a TESCAN MIRA 3 XMU scanning electron microscope (SEM) (Brno, Czech Republic). Proton nuclear magnetic resonance spectroscopy (^1^H-NMR) was performed at room temperature on a JEOL JNM-ECZ400S spectrometer (Tokyo, Japan) at 400 MHz by using chloroform-d as solvent. Melting points were determined using an Electrothermal 9100 apparatus (Staffordshire, UK). The 2,2-diphenyl-1-picrylhydrazyl (DPPH) radical scavenging activity of GO/Chol-ZnPc was examined according to Blois method by using gallic acid as a standard [51]. In a regular measurement, DPPH was mixed with GO/Chol-ZnPc in DMSO for 2 h at room temperature, and the measurements were performed with an enzyme-linked immunosorbent assay (ELISA) reader.

## 3. Results and Discussion

**Figure 3:**
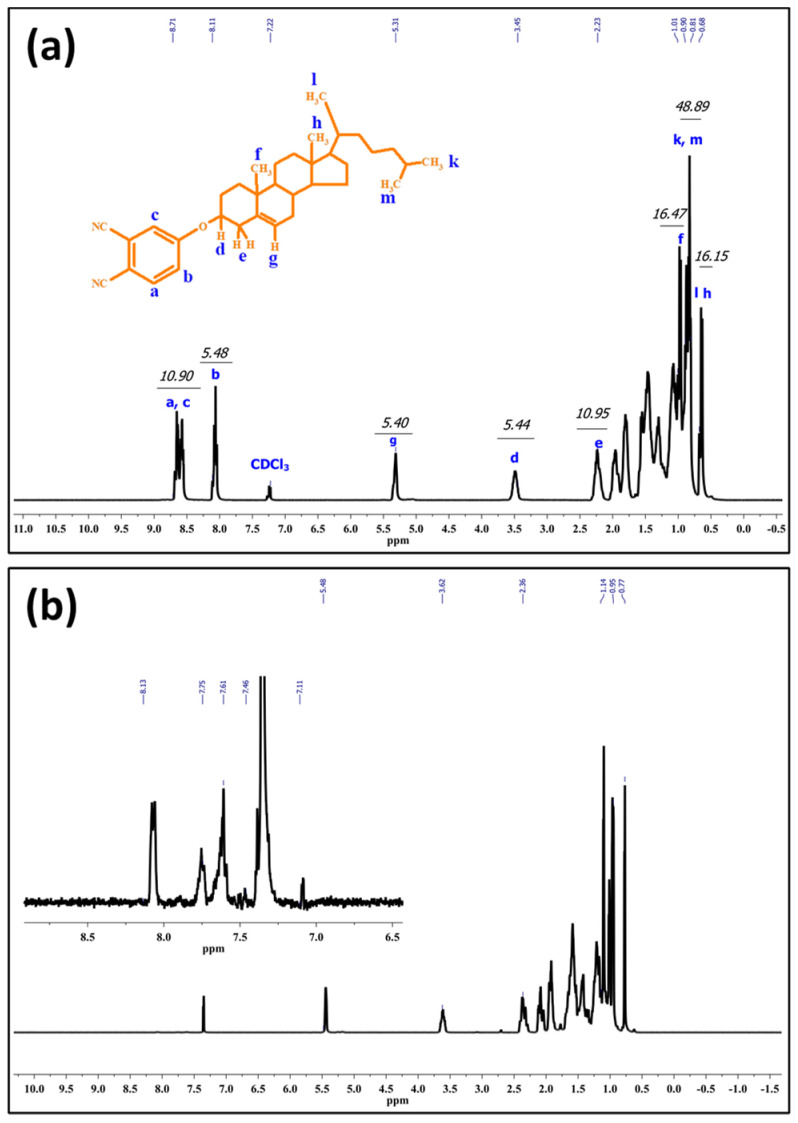
^1^H-NMR spectrum of Chol-phthalonitrile (**a**) and Chol-ZnPc (**b**).

It would be crucial to verify successful formation of Chol-phthalonitrile before the synthesis of Chol-ZnPc. Accordingly, we first studied the ^1^H-NMR spectrum of Chol-phthalonitrile, as shown in Figure 3. The aromatic protons belonging to phthalonitrile group were observed in the range of 8.7–8.1 ppm, as could be expected, while the protons belonging to the Chol group were observed between 5.3 and 0.7 ppm (Figure 3). Further, all relevant bonds are identified in Figure 3, and both the respective positions and integral ratios of the characteristic peaks were in good agreement with previous reports [52,53,54]. Regarding Chol-ZnPc, it was prepared by tetramerization reaction of Chol-phthalonitrile and ZnCl_2_. The ^1^H-NMR spectrum of Chol-ZnPc exhibited aromatic protons on the Pc ring in the range of 8.1–7.1 ppm, and the protons belonging to the Chol group were between 5.5 and 0.5 ppm. These chemical shifts were compatible with the structure of Chol-ZnPc, considering the ^1^H-NMR spectrum of the Chol-phthalonitrile. The chemical shifts in the ^1^H-NMR spectra of both Chol-phthalonitrile and Chol-ZnPc are listed in the experimental section. Overall, ^1^H-NMR results suggested that both Chol-phthalnotitrile and Chol-ZnPc were successfully prepared, and that the as-synthesized compounds were pure.

**Figure 4:**
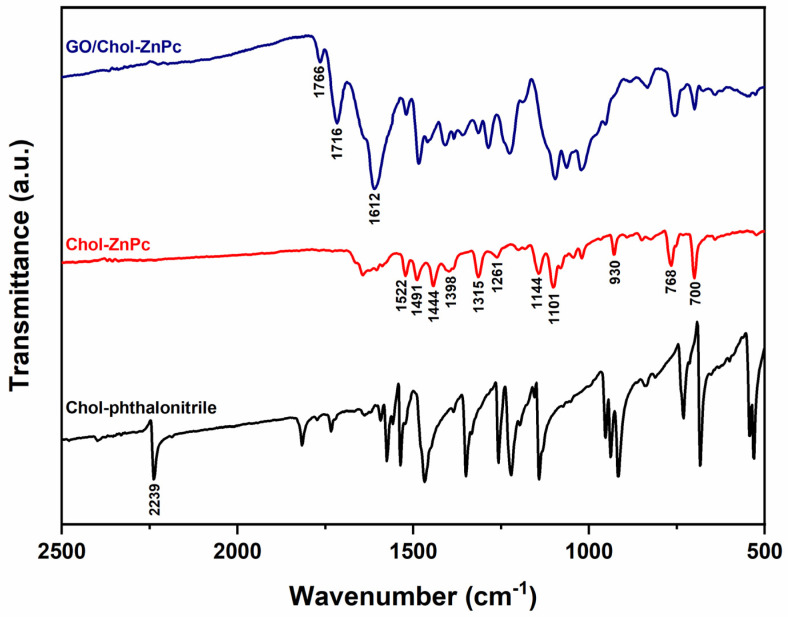
FT-IR spectra of Chol-phthalonitrile, Chol-ZnPc, and GO/Chol-ZnPc.

Incorporation of GO to Chol-ZnPc was verified by FT-IR analysis, as shown in Figure 4. The characteristic C≡N peak of the phthalonitrile was recorded at about 2239 cm^−1^ in the FT-IR spectrum of Chol-phthalonitrile (Figure 4). This peak was not detected in the FT-IR spectrum of Chol-ZnPc, further indicating the successful tetramerization reaction. Also, the IR peaks at about 1522 cm^−1^, 1491 cm^−1^, 1444 cm^−1^, 1398 cm^−1^, 1315 cm^−1^, 1261 cm^−1^, 1144 cm^−1^, 1101 cm^−1^, 930 cm^−1^, 768 cm^−1^, and 700 cm^−1^ could be ascribed to the characteristic skeletal vibrations of ZnPc macrocycles, and were observed in Chol-ZnPc as expected [55,56,57,58,59,60,61,62]. Particularly, the characteristic Pc macrocycle bands at about 1491 cm^−1^, 1101 cm^−1^, and 768 cm^−1^ were red-shifted in the GO/Chol-ZnPc, indicating interactions between GO and Chol-ZnPc. Regarding the peaks at about 1766 cm^−1^ and 1716 cm^−1^, they could be ascribed to the C=O stretching vibrations of carbonyl and carboxyl groups of GO [63,64,65,66]. Moreover, the peak at about 1612 cm^−1^ could be ascribed to the C=C stretching vibrations of graphitic domains in GO [63,64,65]. These three peaks were only observed in the FT-IR spectrum of GO/Chol-ZnPc, and their presence indicates that the GO/Chol-ZnPc composites were successfully prepared.

**Figure 5:**
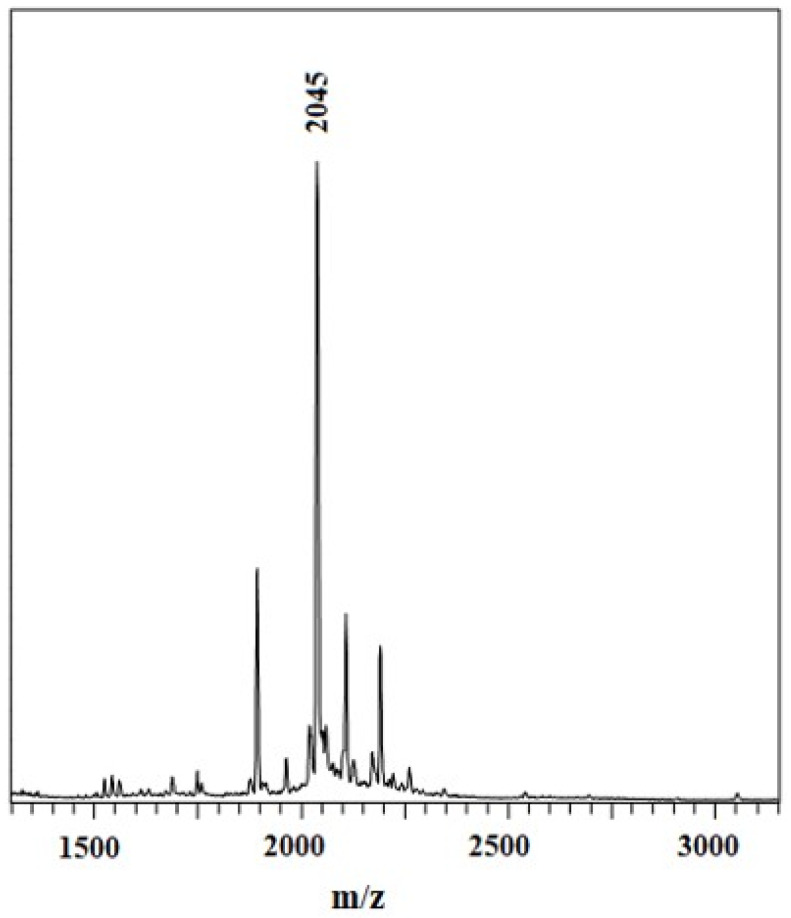
MALDI-TOF MS spectra of Chol-ZnPc.

Further, the elemental analysis and matrix-assisted laser desorption ionization time-of-flight mass spectrometry (MALDI-TOF MS) results are given in the experimental section and Figure 5, and both results verify the successful preparation of the samples.

**Figure 6:**
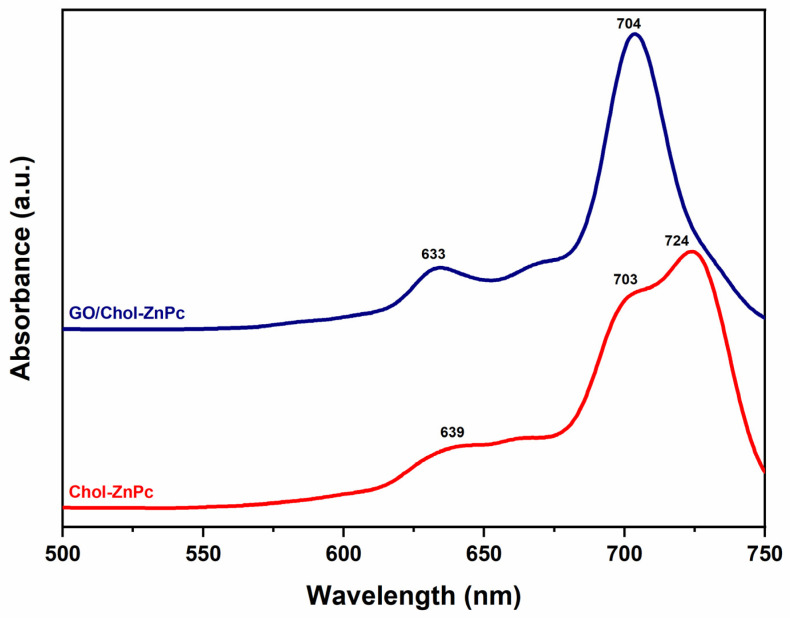
UV–Vis spectra of Chol-ZnPc and GO/Chol-ZnPc.

In addition, Chol-ZnPc and GO/Chol-ZnPc were studied using ultraviolet–visible (UV–Vis) spectroscopy in DMSO (Figure 6). Figure 6 shows that both the Chol-ZnPc and GO/Chol-ZnPc exhibited the typical Q bands of Pcs within the therapeutic window, in accordance with previous reports [67,68,69]. As is known, the characteristic Q band is due to the π−π* transition of the Pc ring from the highest occupied molecular orbital (HOMO) to the lowest unoccupied molecular orbital (LUMO) [70]. Interestingly, while GO/Chol-ZnPc showed monomeric Q bands, Chol-ZnPc exhibited a doublet with absorbance maxima at about 703 nm (Q_x_) and 724 nm (Q_y_), suggesting aggregation of Chol-ZnPc in the solvent [67]. Upon introduction of GO, the doublet disappeared and turned into a single peak, an observation in good agreement with previous reports [37]. Further, this peak was blue-shifted as compared to Q_y_. In fact, most previous studies reported a red shift in the Q band upon covalent attachment of GO to Pc [71,72,73]. However, the processing time was too limited in the present work for a covalent attachment. In addition, it could be realized that the functional groups in the present work cannot allow a hydrogen bonding, unlike the previous work of Yabas et al. [37]. In fact, both the red shift and the blue shift of the Q band were reported as depending on the substituted groups in previous works where various GO and Pc derivatives were coupled at relatively similar processing conditions [42,74,75], and the blue shift of the Q_y_ in the present work might be ascribed to π−π and electrostatic interactions between GO and Chol-ZnPc.

**Figure 7:**
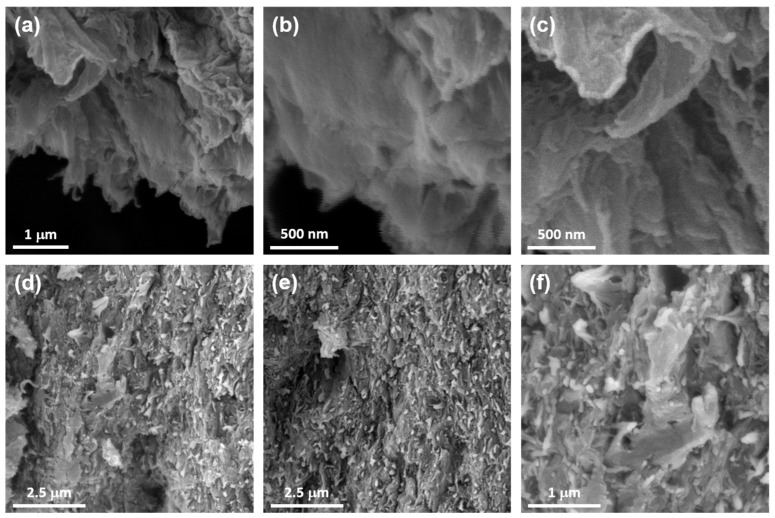
SEM images of GO (**a**–**c**) and GO/Chol-ZnPc (**d**–**f**).

The morphological features of the Chol-ZnPc and GO/Chol-ZnPc were studied using SEM analysis, as shown in Figure 7. Figure 7a–c show that the microstructure of GO exhibited layers of crumpled sheets. Then, upon preparation of GO/Chol-ZnPc, mostly spherical yet nonuniform particles and aggregates of Chol-ZnPc were found to be homogeneously distributed in the composite, as can be seen in Figure 7d–f. Also, the presence of Zn in the Chol-ZnPc was further confirmed by the SEM-EDX, which revealed that the atomic percentage of Zn in the compound was about 2% (Figure S1).

As is known, the efficiency of fluorescence emission is important in PDT applications, particularly for image guidance. In this sense, we studied the fluorescence spectra of Chol-ZnPc and GO/Chol-ZnPc in DMSO, as can be seen in Figure 7, and calculated the $\Phi_{F}$ according to Equation (1) [76]:

(1) $$\Phi_{F}=\Phi_{F_{\left(Std\right)}}\times \frac{F\times A_{Std}\times n^{2}}{F_{Std}\times A\times n_{Std}^{2}}$$

where $\Phi_{F}$, F, A, and n represent the fluorescence quantum yield, area under the fluorescence emission curve, absorbance at the excitation wavelength, and the refractive index of the solvent, respectively. The subscript Std in Equation (1) denotes that the corresponding data are for the standard sample, which was unsubstituted ZnPc ($\Phi_{F}=0.20$ in DMSO [77]) in the present work.

**Figure 8:**
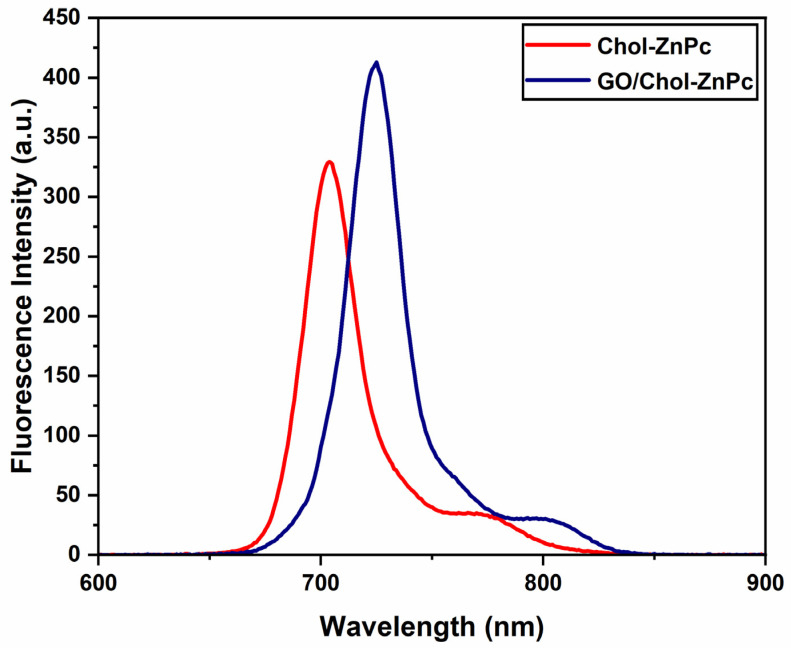
Fluorescence emission spectra of Chol-ZnPc and GO/Chol-ZnPc.

Figure 8 shows that the emission peak was shifted towards higher wavelengths in the GO/Chol-ZnPc composite as compared to the Chol-ZnPc. This could be ascribed to the π–π and electrostatic interactions between GO and Chol-ZnPc in the composite. Also, fluorescence emission was clearly enhanced in the composite (Figure 8), suggesting better charge transport in the GO/Chol-ZnPc. In line with this, $\Phi_{F}$ of the GO/Chol-ZnPc composite ($\Phi_{F}=0.36$) was, indeed, higher than that of both Chol-ZnPc ($\Phi_{F}=0.31$) and unsubstituted ZnPc, as listed in Table 1.

**Figure 9:**
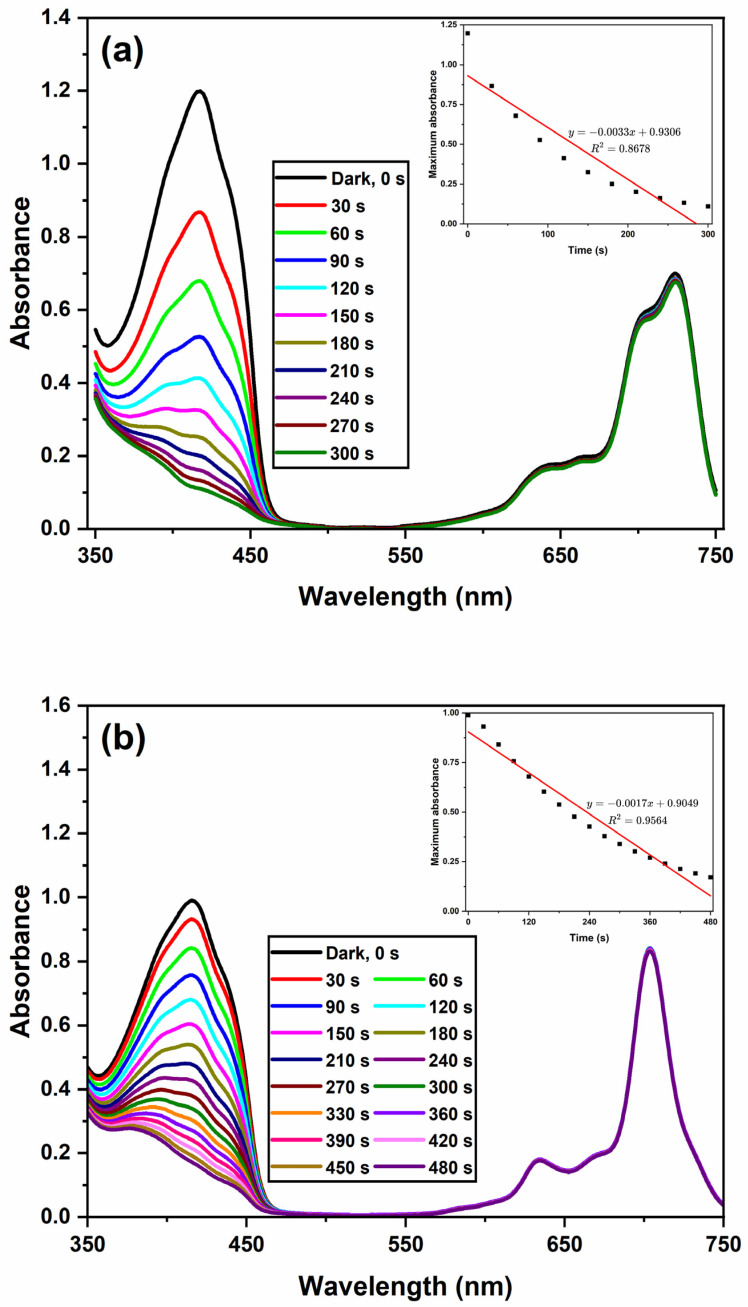
Depletion in the absorbance of DBPF at 417 nm with respect to irradiation time for Chol-ZnPc (**a**) and GO/Chol-ZnPc (**b**). Inset shows the bleaching rate of DBPF at 417 nm upon illumination.

The singlet oxygen quantum yield ($\Phi_{\Delta }$) was determined in DMSO via a common method [77,78,79,80,81,82] by using 3-diphenylisobenzofuran (DPBF) as a singlet oxygen scavenger and unsubstituted ZnPc as a standard. As is known, DPBF does not decompose with singlet oxygen; instead, its reaction with singlet oxygen causes a decrease in the absorbance intensity at 417 nm, which could be used to measure the singlet oxygen generation ability [78]. Thus, DMSO solutions containing Chol-ZnPc and GO/Chol-ZnPc were prepared separately with DPBF in the dark, and irradiated for certain times. Then, the singlet oxygen generation-dependent degradation in the absorbance of DPBF was monitored for both Chol-ZnPc and GO/Chol-ZnPc at each time interval, as shown in Figure 9, and the singlet oxygen quantum yields were calculated by Equation (2) [77]:

(2) $$\Phi_{\Delta }=\Phi_{\Delta }^{Std}\times \frac{R_{DPBF}\times F_{abs}^{Std}}{R_{DPBF}^{Std}\times F_{abs}}$$

where Φ_Δ_, $R_{DPBF}$, and F_abs_ represent the singlet oxygen quantum yield, photobleaching rate, and absorption correction coefficient, respectively. The ratio of $\frac{F_{abs}^{Std}}{F_{abs}}$ is calculated using Equation (3) [78,79,80]:

(3) $$\frac{F_{abs}^{Std}}{F_{abs}}=\frac{1-10^{{-OD}^{Std}}}{1-10^{-OD}}$$

where OD refers to the absorption at the wavelength of light source. The superscript Std in Equations (2) and (3) denotes that the corresponding data are for the standard sample, which was unsubstituted ZnPc ($\Phi_{∆}=0.67$ in DMSO [77]) in the present work.

The results show that Chol-ZnPc exhibited a lower singlet oxygen generation ability (Φ_∆_ = 0.54) than unsubstituted ZnPc, as can be seen in Table 1. However, the extent of this decrease was relatively limited. In addition, the substitution of Chol might enhance targeted delivery of the photosensitizer to cancer cells, because it is known that cancer cells are keen to attract as much Chol as possible through expressing more LDL receptors [34,35,36]. In the field, the encapsulation of Pcs in liposome-based nanoparticles is among the most common approaches for the targeted delivery of photosensitizers [16,17,18,19,20,21,22]. In this approach, the photosensitizers were released during the lipid exchange with lipoproteins, they were attached to lipoproteins, and such attachment, particularly to LDLs, helps to the selective targeting of cancer cells [23,24,25,26,27,28,29]. In this sense, the presence of Chol might provide improved binding to LDL for better drug delivery, considering that Chol-modified structures could easily interact with Chol-rich molecules [36,83,84,85,86]. This means that the overall PDT performance might be enhanced in Chol-ZnPc via a possible improvement in the photosensitizer uptake of cancer cells, despite the slight decay in Φ_∆_. In addition, the decrease in Φ_∆_ could actually be neutralized by the introduction of GO to Chol-ZnPc, as can be seen in Table 1. In fact, the Φ_Δ_ of GO/Chol-ZnPc (Φ_∆_ = 0.78) was actually higher than that of both Chol-ZnPc and unsubstituted ZnPc (Table 1). This means that a significant enhancement was realized upon introduction of GO, an improvement that might be ascribed to the better charge transport properties in the composite. Furthermore, this enhancement in Φ_Δ_ was reached simultaneously with an increase in Φ*_F_*, as shown in Table 1. This is particularly important as it is not an easy task to concurrently realize a high Φ*_F_* and Φ_Δ_, since a high Φ_Δ_ is often accompanied with a low Φ*_F_*, considering that intersystem crossing competes with fluorescence [42,67,82,87,88,89,90,91,92,93,94,95]. Strikingly, as compared to the Φ*_F_* and Φ_Δ_ results of previous similar studies in DMSO, the as-prepared GO/Chol-ZnPc composite in the present work seems to be a promising candidate, considering that Gunsel et al. [45] reported a Φ*_F_* of 0.092 and Φ_Δ_ of 0.628 for a 10% GO containing ZnPc derivative, Gunsel et al. [44] reported a Φ*_F_* of 0.186 and Φ_Δ_ of 0.638 for a 10% GO containing ZnPc derivative, Matshitse and Nyokong [96] reported a Φ*_F_* of 0.09 and Φ_Δ_ of 0.11 for a 50% graphene quantum dot containing ZnPc derivative, Nwahara et al. [97] reported a Φ*_F_* of 0.23 and Φ_Δ_ of 0.52 for a 23% glutathione-capped graphene quantum dot containing aluminum Pc derivative, and Nwahara et al. [98] reported a Φ*_F_* of 0.16 and Φ_Δ_ of 0.65 for a 33% L-glutathione functionalized graphene quantum dot containing ZnPc derivative.

**Figure 10:**
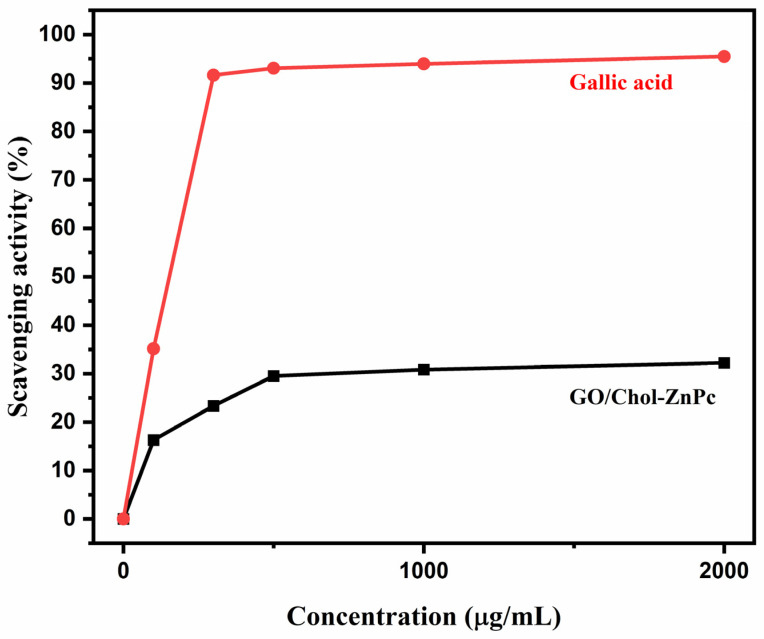
DPPH free radical scavenging activity of GO/Chol-ZnPc and gallic acid standard.

On the other hand, it is worth mentioning that the actual PDT performance of a photosensitizer on a living cell is a much more complex case, and it necessitates information on pharmacodynamics and pharmacokinetics to draw more precise conclusions. Particularly, although some previous reports indicated positive effects of using GO in terms of improving PDT properties [41,42,43,44,45,46,47,48,99], there exists diverse information regarding the cytotoxicity of GO depending on the synthesis conditions, functional groups, aggregation, contamination, cell type, and so forth [100,101,102,103,104,105,106,107]. While we did not provide the cytotoxicity assay of the GO/Chol-ZnPc in the present work, the antioxidant activity of the composite was evaluated using the Blois method, as shown in Figure 10. This method is based on the reduction of DPPH, a stable free radical, by the antioxidant and its color change. As is known, DPPH provides the maximum absorption at 517 nm (violet color) [108,109]. When the single electron in the DPPH free radical pairs with hydrogen from the antioxidant, the intensity of the strong absorbance band at 517 nm decreases, and the color of DPPH changes from violet to yellow. We determined the color intensity of the component using an ELISA reader, which helps to observe the antioxidant property of the material [110,111]. Also, the antioxidant activity of gallic acid, a typical antioxidant, was determined at the same conditions, and is plotted in Figure 10 for comparison. For GO/Chol-ZnPc, only a slight change in the violet color of DPPH was observed after 2 h. Thus, as can be seen in Figure 10, the antioxidant activity of the GO/Chol-ZnPc composite was much lower than that of the gallic acid standard, and the percentage antioxidant activity of the composite was less than 50% at all concentrations tested. Hence, it can be said that GO/Chol-ZnPc does not show a significant antioxidant activity on the DPPH radical. Overall, the GO/Chol-ZnPc composite exhibited relatively high Φ*_F_* and Φ_Δ_ simultaneously, did not exhibit a significant antioxidant activity, and might be considered as a promising material for PDT applications.

## 4. Conclusions

The present work involves preparation of Chol-ZnPc and GO/Chol-ZnPc, and investigation of their PDT properties. Briefly, Chol-phthalonitrile was synthesized first, and then Chol-ZnPc was prepared through the tetramerization reaction of Chol-phthalonitrile and ZnCl_2_. Following this, GO/Chol-ZnPc composites were prepared using the self-assembly method. The samples were characterized by ^1^H-NMR, FT-IR, UV–Vis, SEM, DPPH, MALDI-TOF MS, and elemental analysis. Further, fluorescence quantum yield and singlet oxygen quantum yield of the Chol-ZnPc and GO/Chol-ZnPc were also determined for PDT applications. The results show that Chol-ZnPc exhibited a slightly lower singlet oxygen generation ability (Φ_∆_ = 0.54) than unsubstituted ZnPc. However, it is likely that Chol substitution might improve the photosensitizer uptake of the cancer cells, considering that the encapsulation of photosensitizers to liposome-based nanoparticles is a common drug targeting strategy in the field. This is because it is known that cancer cells attract more Chol than normal for proliferation by expressing high LDL receptors, and the Chol substitution might provide a better binding between the photosensitizer and LDL following the release of the Pcs during lipid exchange of liposomes with lipoproteins in the targeted delivery of photosensitizers by using liposome-based nanoparticles. Also, we report that preparation of GO/Chol-ZnPc resulted in the simultaneous enhancement of Φ*_F_* and Φ_Δ_ to 0.36 and 0.78, respectively. This is particularly important as a combination of high Φ*_F_* and Φ_Δ_ is desired for image-guided PDT applications, despite a high Φ*_F_* often being an indicator of low Φ_Δ_. In addition, the antioxidant activity of the GO/Chol-ZnPc composite was also determined using DPPH radical scavenging assay, considering the diverse information in the literature regarding the cytotoxicity of GO, and the results of the DPPH tests suggest that GO/Chol-ZnPc did not exhibit a significant antioxidant activity. Overall, it could be thought that GO/Chol-ZnPc composites might have the potential to provide combined advantages for PDT applications, particularly in terms of image guidance and singlet oxygen generation.

## Figures and Tables

**Table 1:** PDT performance of Chol-ZnPc and GO/Chol-ZnPc in DMSO.

Compounds	$\Phi_{F}$	$\Phi_{∆}$
Chol-ZnPc	0.31	0.54
GO/Chol-ZnPc	0.36	0.78
Unsubstituted ZnPc	0.20 ^a^	0.67 ^a^

^a^ [77].

## Data Availability

Data will be made available on request.

## Supplementary Materials

The following supporting information can be downloaded at: https://www.mdpi.com/article/10.3390/ma16227060/s1, Figure S1. SEM-EDX spectrum of Chol-ZnPc.
