# Supplementary figures and images for: Graphene Oxide/Cholesterol-Substituted Zinc Phthalocyanine Composites with Enhanced Photodynamic Therapy Properties
Source: Materials (Basel). 2023 Nov 7;16(22):7060. doi: 10.3390/ma16227060 (PMC10672206; doi:10.3390/ma16227060)

## Supplementary Material

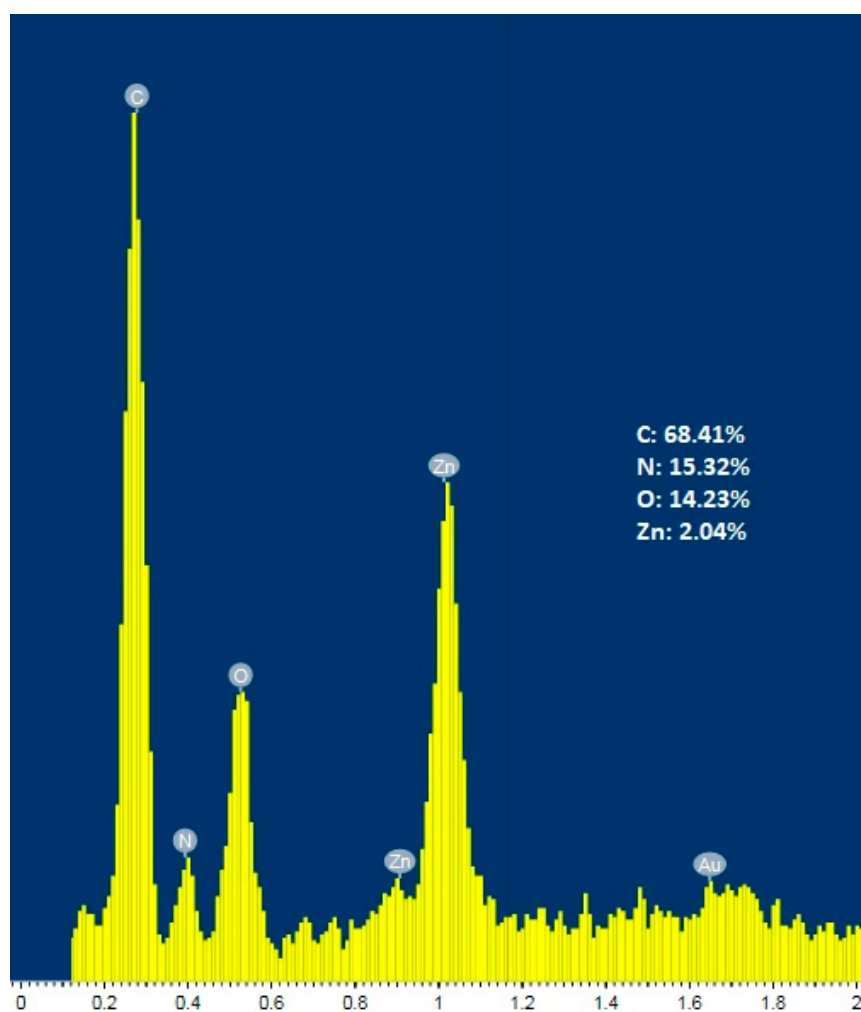

Figure S1. SEM-EDX spectrum of Chol-ZnPc.

Supplement: Supplementary file 1 [file materials-16-07060-s001.zip › materials-2636262-supplementary.pdf]
